# The Interplay Between Lymphatic Vessels and Chemokines

**DOI:** 10.3389/fimmu.2019.00518

**Published:** 2019-04-12

**Authors:** Rae H. Farnsworth, Tara Karnezis, Simon J. Maciburko, Scott N. Mueller, Steven A. Stacker

**Affiliations:** ^1^Tumor Angiogenesis and Microenvironment Program, Peter MacCallum Cancer Centre, Melbourne, VIC, Australia; ^2^Sir Peter MacCallum Department of Oncology, The University of Melbourne, Parkville, VIC, Australia; ^3^Lymphatic and Regenerative Medicine Laboratory, O'Brien Institute Department, St. Vincent's Institute of Medical Research, Fitzroy, VIC, Australia; ^4^Department of Microbiology and Immunology, Peter Doherty Institute for Infection and Immunity, The University of Melbourne, Melbourne, VIC, Australia; ^5^The Australian Research Council Centre of Excellence in Advanced Molecular Imaging, Melbourne, VIC, Australia; ^6^Department of Surgery, Royal Melbourne Hospital, The University of Melbourne, Parkville, VIC, Australia

**Keywords:** chemokine, lymphatics, endothelial, chemokine receptor, lymphangiogenesis, lymphatic remodeling

## Abstract

Chemokines are a family of small protein cytokines that act as chemoattractants to migrating cells, in particular those of the immune system. They are categorized functionally as either homeostatic, constitutively produced by tissues for basal levels of cell migration, or inflammatory, where they are generated in association with a pathological inflammatory response. While the extravasation of leukocytes via blood vessels is a key step in cells entering the tissues, the lymphatic vessels also serve as a conduit for cells that are recruited and localized through chemoattractant gradients. Furthermore, the growth and remodeling of lymphatic vessels in pathologies is influenced by chemokines and their receptors expressed by lymphatic endothelial cells (LECs) in and around the pathological tissue. In this review we summarize the diverse role played by specific chemokines and their receptors in shaping the interaction of lymphatic vessels, immune cells, and other pathological cell types in physiology and disease.

## Introduction

Cells in complex vertebrates receive signals from extracellular environment which coordinate a raft of important cellular programs and functions (1). These signals can be through direct cell-to-cell contact or by the use of soluble molecules synthesized and secreted by neighboring or distant cells. Growth factors and cytokines are examples of soluble proteins that have potent cellular effects through designated cell surface receptors, such as growth and differentiation (2). A subset of the cytokine proteins that act to induce the movement of cells are the chemokines (*-kinos* from the Greek for movement) (3). Chemokines are small, highly conserved polypeptides of 70–100 amino acids. While having a conserved three-stranded β-sheet/α-helix tertiary structure they are divided into several subfamilies (CXC, CC, XC, and CX3C) based on variations in their quaternary structure and critical cysteine residues (4, 5). They exert their effects through cell surface G-protein coupled receptors on target cells (4) that can act as homo- or heterodimers depending on the context. This family has now expanded to include at least 51 chemokines and 20 receptors, plus (presently) four atypical or decoy receptors which typically dampen chemokine activity by binding and internalizing chemokines without initiating G-protein-dependent signaling (5–7).

Chemokines act by establishing gradients to direct random or directed migration of cells bearing cognate receptors from lower to higher concentrations of ligands. These gradients are often formed through the interaction with proteoglycans attached to the cell surface or extracellular matrix. Diversity within the chemokine system is generated both structurally and functionally through an array of different receptors and ligands with precise or promiscuous binding affinity, where splice variants, post-translational modifications including nitrosylation, citullination, and many forms of proteolytic cleavage (8) can all diversify signaling leading to events that are either chemoattractive or chemorepulsive (5, 9, 10). The biological effects of the chemokine family are broad-ranging as they can be used to move individual cells, subsets of cells or large groups of cells in order to achieve the outcomes of significant processes such as immune cell development, embryogenesis, angiogenesis, phagocytosis and survival/apoptosis (5). Expand this to controlling these cell population during infection, immunity, inflammation, and other pathologies and the extensive roles of chemokines in the mammal is clear.

The movement of cells in normal and pathological situations is highly dependent on the circulatory system, which allows long and short range transport, and exit and entry from all tissues. Previous studies have shown the critical role of blood vessels in chemokine action, in particular directing key cellular effectors of the immune response (11). Blood and lymphatic vessels work together to control fluid and cells in the circulation and tissues, yet the blood vessels have often received the most attention. However, the important and independent roles the lymphatics play in cellular interactions in normal physiology, development, and pathology are becoming evident through studies in a number of areas highlighting the organ- and subtype- specific activity of lymphatic vessels (12–14).

Lymphatic vessels have gained a greater prominence in our thinking over the past two decades as molecular tools have facilitated clear discrimination from blood vessels (15–17). Further, the characterization of factors required for growth and differentiation of lymphatic endothelial cells (LECs) *in vitro* has provided a more in-depth understanding of their unique biological function and differences to blood vascular endothelium (18). Extensive *in vivo* studies using promoters with specificity to the lymphatic compartment has also identified key functional roles for the lymphatics and LECs in development and disease (14, 19), and other functional screens have highlighted the unique features of LECs (20, 21). These unique responses of lymphatic vessels are often regulated through the interaction of cells and signaling molecules with the LECs lining the lumens of lymphatic vessels.

**Figure 1:**
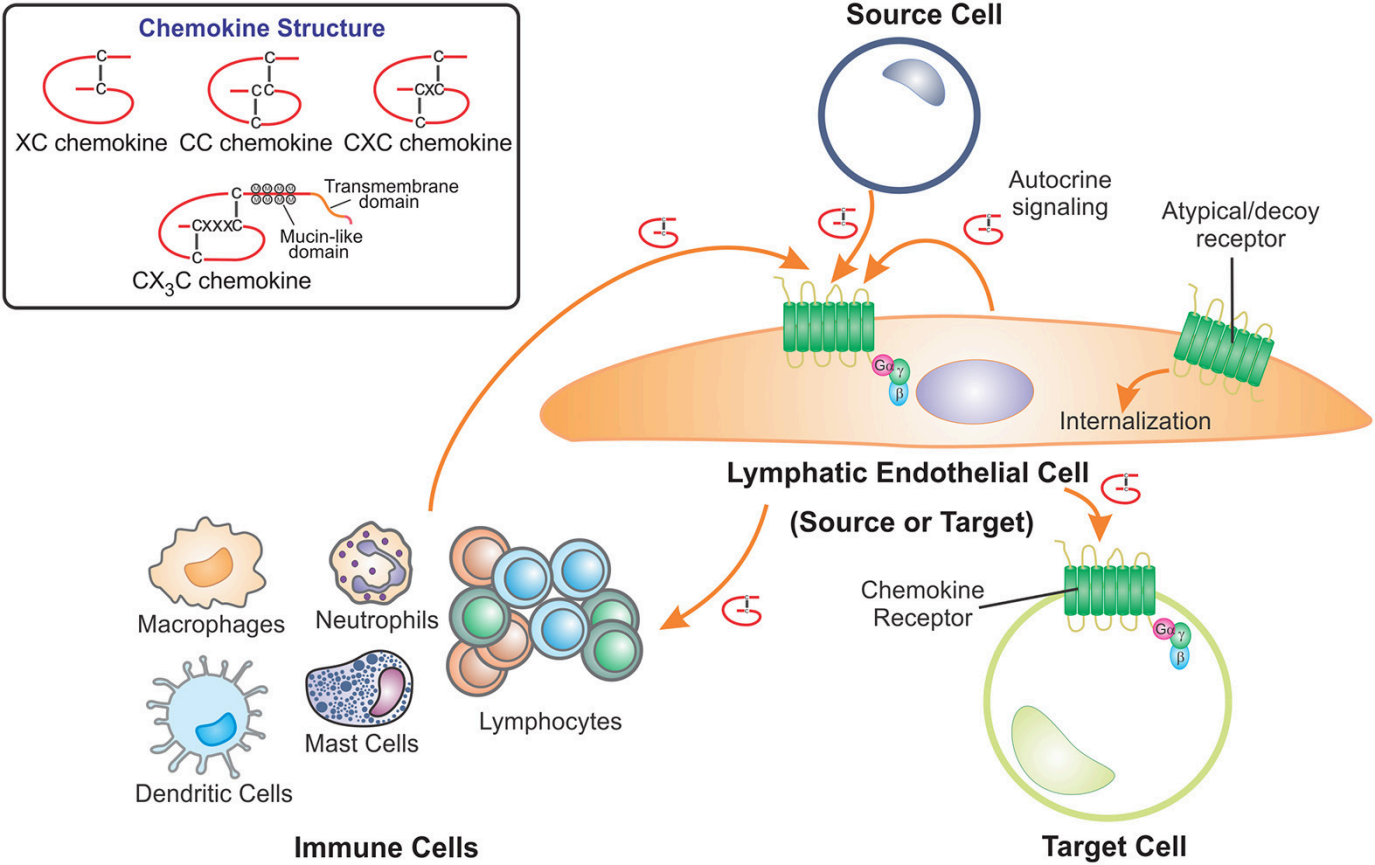
LECs contribute to the role of chemokines by acting as either the source or target cell. Chemokines are a structurally related family of cytokines that direct cell movement and are classified as XC, CC, CXC, or CX_3_C chemokines depending on the positions of conserved cysteine residues which form disulfide bridges. Lymphatic endothelial cells (LECs) receive chemokine signals, using cell surface G protein-coupled receptors, from source cells which include cells of the immune system, other LECs, fibroblasts and pathological cells types such as cancer cells. LECs also act as the source of chemokines, secreting these proteins to act on chemokine receptors present on other target cells. Furthermore, LECs can regulate chemokine availability by scavenging and internalizing secreted chemokines via atypical or decoy chemokine receptors.

The paradigm of chemokine action involving the lymphatics is potentially complex. The lymphatics can both be the source of the chemokines, express the receptors, or both (Figure 1; Table 1). As a vessel for the passage of many circulating cells lymphatics also act as a conduit allowing the flow of chemokines or cells to other targets; for instance to lymph nodes (LNs). Akin to the action of chemokines in blood vessel function the lymphatics provide a surface for the attraction and interaction of immune cells in pathological contexts (3, 63, 64). This review aims to highlight the interplay between lymphatic vessels and chemokines in a range of biological contexts from embryonic development through to regulation of immunity and a range of human pathologies.

**Table 1:** Chemokine-mediated cellular interactions with LECs.

**CHEMOKINE LIGANDS EXPRESSED BY LECs**				
**Ligand**	**Receptor**	**Chemokine target cells**	**Biological context**	**References**
CCL21	CCR7	Activated DCs, T lymphocytes, neutrophils	Immune cell trafficking into initial lymphatics and to/within LNs	(22–24)
		LTi cells	Lymphoid organogenesis	(25)
		Tumor cells (various)	Lymphogenous tumor metastasis	(11, 26)
CCL27	CCR10	Skin-homing T cells, LEC subset	T cell trafficking in precollecting lymphatics	(27)
CXCL10	CXCR3	Macrophages	Upregulated in type 2 diabetes	(28)
		Tumor cells (Colorectal, melanoma)	LN metastasis	(29, 30)
CCL20	CCR6	DCs, T and B cell subsets	Immune cell trafficking to LN	(31, 32)
CXCL12	CXCR4	DCs	Immune cell trafficking to LN	(33)
		Tumor cells (various)	LN metastasis	(34–37)
CXCL1	CXCR2	Tumor cells (Gastric)	Invasion and metastasis	(38, 39)
CCL2	CCR2	Macrophages	Developmental lymphangiogenesis	(40)
CX3CL1	CX3CR1	DCs	Immune cell trafficking to LN	(41)
CCL5	CCR5	Tumor cells (Breast)	Metastatic niche formation, metastasis	(42)
CCL1	CCR8	Tumor cells (Melanoma)	LN metastasis	(43)
**CHEMOKINE RECEPTORS EXPRESSED BY LECs**				
**Receptor**	**Ligand/s**	**Chemokine source cells**	**Biological context**	**References**
CCR10	CCL27	Keratinocytes, tumor cells	LEC migration in tumor lymphangiogenesis, lymphatic patterning	(44)
	CCL28	Mucosal epithelia, tumor cells	LEC migration *in vitro* and *in vivo*	(44)
CXCR4	CXCL12	Embryonic arteries	Developmental lymphatic patterning	(45)
		Tumor cells and stroma	Tumor lymphangiogenesis, lymphogenous metastasis	(46)
CXCR2	CXCL5	Melanoma cells	Tumor lymphangiogenesis, lymphogenous metastasis	(47)
	CXCL1	Gastric cancer LECs	Tumor lymphangiogenesis, lymphogenous metastasis	(38)
	CXCL8	Overexpressed	Experimental lymphedema	(48)
**ATYPICAL CHEMOKINE RECEPTORS EXPRESSED BY LECs**				
**Receptor**	**Ligand/s^a^**	**Chemokine source cells**	**Biological context of receptor**	**References**
ACKR1 (DARC)	CCL2, CCL5, CCL7, CCL11, CCL13, CCL14, CCL17, CXCL1, CXCL2, CXCL3, CXCL5, CXCL6, CXCL7, CXCL8, CXCL11	Various	Precollecting LECs	(27)
ACKR2 (D6, CCBP2)	CCL2, CCL3, CCL3L1, CCL4, CCL4L1, CCL5, CCL7, CCL8, CCL11, CCL12, CCL13, CCL14, CCL17, CCL22, CCL23, CCL24	Various	Afferent lymphatics in various tissues Developmental lymphatic patterning Immune cell trafficking in inflammation/immunity Tumor lymphatics Vascular tumors Kaposi sarcoma	(49–51) (40) (52, 53) (51, 54–57) (51) (58)
ACKR3 (CXCR7)	CXCL11, CXCL12	Various	Increased expression during renal allograft rejection	(59)
	Adrenomedullin		Developmental lymphatic patterning	(60)
ACKR4 (CCRL1)	CCL19	Dermal stromal cells	Immune cell trafficking to LN	(61)
	CCL21	Fibroblastic reticular cells, LECs	DC trafficking into LN parenchyma	(62)
	CCL25, CXCL13	Lymphoid stromal cells	Immune cell trafficking; direct interaction with LEC-expressed ACKR4 not studied	(49)

a *Compiled from references (5) and (49)*.

## Development

### Regulation of Lymph Node Organogenesis

In accordance with their function in providing immune surveillance for particular organs or regions of tissue, LNs develop at strategic locations along the vasculature, typically at the branch points of large veins (25, 65). Although the mechanisms controlling the precise location and subsequent assembly of LNs are incompletely understood, recent evidence suggests multiple roles for lymphatics as well as the venous vasculature.

Chemokines are already known to be critical for initiating the development of LNs and other secondary lymphoid organs such as the Peyer's patches in the gut (25). Mice lacking CXCL13 or its receptor CXCR5 fail to develop particular subsets of peripheral LNs and also exhibit impaired Peyer's patch formation (66, 67). Combined deficiency of CXCR5 and CCR7 completely ablated the formation of peripheral LNs (68), although interestingly deficiency of CCR7 alone had only a mild impact (68, 69). In the prevailing model of LN development, lymphoid tissue organizer (LTo) cells—cells of mesenchymal origin induced by neuronally-derived retinoic acid signaling (70) at putative sites of LN development—secrete the chemokines CXCL13, CCL19, and CCL21 which in turn recruit CXCR5- and CCR7-expressing haematopoietic lymphoid tissue initiating (LTi) cells into the growing LN anlage (25, 65, 68). LTi cells extravasate from veins at junctions where smooth muscle coverage is sparse (71) and make contact with LTo cells, whereupon a positive feedback loop ensues: activation of IL-7Rα on LTi cells by LTo-expressed IL-7 upregulates lymphotoxin expression by LTi cells, which in turn promotes further chemokine secretion by LTo cells (66, 72). These reciprocal interactions lead to the expansion of both cell populations, growth of the LN anlage and subsequent differentiation of lymphoid organ subcompartments (25, 65). However, the persistence of LN formation when LTβR signaling was specifically ablated in CXCL13 or CXCL19-expressing mesenchymal cells (73, 74) suggested that additional LTo cell types may exist.

The contribution of lymphatics to LN organogenesis was previously unclear—LN development is still initiated in mice that lack lymphatics due to global or Tie2 promoter-restricted *Prox1* knockout, although the anlagen in these animals show defects in the differentiation and organization of mesenchymal LTo cells, and are often reduced in size (75). Recent studies utilizing cell type-specific gene knockout approaches have now elaborated multiple roles for lymphatics in LN formation. Lymphatic vessels contribute to early LN initiation by delivering recirculating LTi cells from peripheral tissue to the LN anlage through CCL21/CCR7-mediated chemotaxis (71, 74). The same sphingosine-1-phosphate (S1P) signaling that regulates lymphocyte egress in adult LNs retains LTi cells at the LN anlage, potentiating the molecular crosstalk between LTo and LTi cells that results in further chemokine-mediated LTi recruitment and subsequent LN maturation (25, 74). Peripheral LN anlagen typically form near major venous junctions which run parallel with collecting lymphatic vessels. LECs within the collecting vessel adjacent to the accumulation of LTi and LTo cells subsequently proliferate in a VEGF-C/VEGFR-3-dependent manner to form a disc which eventually expands to envelop the growing LN (71, 76). Functional lymph flow also appears to be essential for complete LN formation as it generates interstitial fluid force which likely stimulates CXCL13 expression by fibroblastic LTo cells (71). Notably, many of the cellular mechanisms and signaling pathways (including chemokines) involved in LN organogenesis are recapitulated in the development of tertiary lymphoid organs in response to pathological insult (25). This suggests that enhanced lymphangiogenesis and lymph flow may contribute to the *de novo* development of lymphoid organs in order to strengthen local immune responses, and that this mechanism may be therapeutically manipulable (25).

### Regulation of Lymphatic Vascular Patterning

Where chemokines mediate interactions between the blood and lymphatic vasculature and other cell types, the endothelial cells lining these vessels are commonly characterized as the source of the chemokine ligand, or the surface to which it binds. However, endothelial cells themselves also express chemokine receptors and can respond to chemokine gradients generated by other cell types. As such, a growing list of chemokines and their receptors have been implicated in directing the growth and patterning of blood and lymphatic vasculature.

CXCR4 and its ligand CXCL12 have well-described roles in promoting angiogenesis and patterning the embryonic vasculature (77–81). Recently their role in patterning the lymphatic vasculature has also been described (45). In zebrafish, expression of *cxcr4a* and *cxcr4b* was detected in lymphatic progenitors sprouting from the posterior cardinal vein, as well as in the developing parachordal line, intersegmental lymphatic vessels, and other large trunk lymphatics such as the thoracic duct (45). Loss- and gain- of function experiments confirmed that these receptors were required for the development of the large trunk lymphatics. Accordingly, dynamically regulated expression of ligand-encoding genes *cxcl12a* and *cxcl12b* in the dorsal aorta and arterial intersomitic vessels directed the parallel migration of the growing lymphatic vessels along these paths (45). Interestingly, another group has shown that upregulation of *cxcl12a* was mediated by the microRNA miR-126, which also synergises with Flt4 (VEGFR-3) signaling (82).

Atypical or decoy chemokine receptors also play important roles in shaping developmental lymphangiogenesis. Mice deficient in ACKR3 (formerly known as CXCR7) exhibit defects in lymphatic development, typified by precocious development of lymph sacs and hyperplasia (60). This phenotype was found to be caused by excessive pro-proliferative signaling from adrenomedullin, a non-chemokine ligand for ACKR3 which is a positive regulator of lymphangiogenesis. Deficiency of ACKR2 (formerly D6; CCBP2, chemokine-binding protein 2) in mice results in hyper-branched lymphatics (40). ACKR2 scavenges LEC-expressed CCL2, which is chemotactic for monocytes via CCR2 signaling, thereby reducing the accumulation of macrophages in proximity to developing lymphatics (40). These macrophages deliver lymphangiogenic growth factors and play important roles in shaping developmental lymphangiogenesis (83, 84). These studies highlight the complex mechanisms by which chemokines orchestrate multiple cellular interactions within the developing embryo.

## Leukocyte Trafficking in Inflammation and Immunity

Arguably the best-characterized chemokine-mediated functions involving the lymphatics are those that regulate trafficking of leukocytes in physiological homeostasis and during inflammation and immune responses. Leukocytes in the peripheral interstitium typically enter initial lymphatics in the first instance, and subsequently migrate through the local plexus of pre-collecting lymphatics before entering the large collecting lymphatic vessels that pump lymph and cells over long distances to LNs, where encounters between antigen-presenting cells and cognate T and B lymphocytes are coordinated (49, 85, 86).

### Entry of Leukocytes Into Peripheral Lymphatic Vessels

CCL21, constitutively expressed by peripheral LECs, has a prominent role in trafficking CCR7-expressing dendritic cells (DCs) through afferent lymphatic vessels to LNs along with other CCR7-expressing cells such as T cell subsets and neutrophils (22–24). Notably, in the peripheral vasculature CCL21 expression is relatively specific to the endothelial cells of initial lymphatics; it is generally absent from blood vascular endothelial cells (BECs) with the exception of high endothelial venules in the LN (23). The elongated, positively-charged C terminus of CCL21 mediates strong binding affinity to diverse proteoglycans as well as collagen IV, allowing it to form chemotactic gradients on the surface of LECs and adjacent extracellular matrix (87). The requirement of CCL21-CCR7 interactions for trafficking immune cells through afferent lymphatics has been long recognized and has been reviewed extensively elsewhere (23, 49), but recent studies continue to shed light on the precise mechanisms and additional chemokines that are involved.

Although low levels of cellular trafficking occur under homeostatic conditions, this increases dramatically during immune responses (49, 86). Accordingly, constitutive expression of homeostatic chemokines in LECs is supplemented during inflammation by increased expression of these chemokines, along with additional “inflammatory” chemokines that shape the immune response. CCR7 is upregulated in DCs by inflammatory stimuli such as TNF-α, while the same stimuli increase CCL21 release by LECs by upregulating transcription and by releasing intracellular stores of the chemokine (88, 89). ACKR4 (previously known as CCRL1) expressed in dermal LECs and keratinocytes plays an essential role in properly directed egress of DCs from skin during inflammation by scavenging the more soluble CCR7 ligand CCL19, which would otherwise retain DCs in skin (61). LEC-expressed ACKR2 also regulates DC egress during inflammation by scavenging inflammatory chemokines to ensure preferential presentation of CCL21 on the cell surface of LECs. This in turn supports adhesion of mature CCR7+ DCs to LECs and their transport to LNs, in preference to immature DCs and inflammatory myeloid cells (52, 53). In mice lacking ACKR2, elevated presentation of inflammatory chemokines such as CCL2 on peripheral and LN LECs leads to congestion of lymphatics by myelomonocytic cells, with downstream impairment of lymphatic transport and consequently dampened antigen-specific immune responses (52). Similar roles for ACKR2 in orchestrating cell migration and resolving the inflammatory response have been described in a range of pathological contexts (50).

DC migration toward lymphatic vessels is also mediated by expression of CXCR4 in activated DCs and its ligand CXCL12 in LECs, although DCs seem to preferentially migrate toward CCL21 when both chemokines are present, indicating a coordinated rather than additive function (33). CX3CL1, an atypical chemokine possessing a transmembrane domain, is upregulated in LECs by TNF-α and mediates basolateral-to-apical migration of CX3CR1-expressing DCs through lymphatic endothelium and DC trafficking to LNs during dermal contact hypersensitivity responses *in vivo* (41). Interestingly, this chemokine is predominately shed from the basolateral surface of LECs by ADAM10 and ADAM17 metalloproteases, in contrast to remaining membrane-bound and behaving as a leukocyte adhesion molecule in BECs (41).

DCs commonly enter lymphatics through binding to immobilized CCL21 puncta specifically localized between the button-like intercellular junctions characteristic of initial lymphatics (90, 91). Direct contact between DCs and LECs also dynamically triggers localized release of CCL21 from within the trans-Golgi network of LECs, further potentiating transendothelial migration (92). Once inside lymphatic vessels, DCs crawl in a semi-directed manner within the flattened lumen, moving in multiple directions but ultimately following an intralymphatic gradient of CCL21 that is generated by lymphatic flow (91, 93). In this context, CCL21 immobilized on the LEC surface mediates not only chemotaxis but also adhesion. Although most DCs and other leukocytes exiting the periphery are thought to enter the initial lymphatics, specific chemokines may regulate entry of particular cell types into other segments of the lymphatic vasculature. CCL27 was found to be specifically expressed in pre-collecting lymphatics, where it promoted the attraction of CCR10+ T lymphocytes *in vitro* and *in vivo* (27). Pre-collecting LECs were also found to overexpress CCL27, CXCL12, CXCL14, and the promiscuous CC chemokine decoy receptor ACKR1 (formerly DARC, Duffy antigen receptor for chemokines) compared to initial LECs, whereas CCL21 was more abundantly expressed in initial LECs (27). The same study found that pre-collecting and initial LECs in the adult human dermis could be discriminated by flow cytometry and immunofluorescence according to their expression levels of Podoplanin—pre-collecting LECs being designated as Podoplanin^low^ and initial LECs Podoplanin^high^ (27). The correspondence between Podoplanin and CCL21 expression levels between LEC subtypes may relate to the ability of the glycoprotein Podoplanin to bind and present CCL21 on the LEC cell surface (94). More remains to be understood about the specific immune cell types which are selectively recruited to initial vs. pre-collecting lymphatics, and the functional importance of these differences.

### Chemokine Signaling Within Lymph Nodes

Once cells pass into collecting lymphatics the lymph flow rate accelerates, and cells are transported passively to LNs where they are delivered into the subcapsular sinus (SCS) (91, 93). Here chemokine gradients also are important in regulating migration and localization of different cell types in the LN parenchyma (49, 85). It has recently been demonstrated that the LECs comprising the LN SCS and medullary lymphatic sinuses have distinct expression profiles, including differential expression of several chemokines and receptors (95). ACKR4 is specifically expressed in LECs of the SCS “ceiling” where it plays an important role in scavenging and internalizing CCL21 to create a gradient that directs DC migration toward and ultimately through the SCS floor into the LN parenchyma (62). CCR7+ T cells arriving through afferent lymphatics have been observed to enter the LN parenchyma preferentially through medullary sinuses, but will transmigrate through the SCS floor only in conjunction with local changes induced by DCs (96). CCL21 is also produced abundantly by fibroblastic reticular cells (FRCs), which constitute the majority of the LN stroma and guide interactions between DCs and naïve T cells both structurally and chemically (97). Notably, lymphatic flow upregulates CCL21 expression by FRCs (98), reiterating the importance of the lymphatics for maintaining proper immune function in the LN microenvironment. Some DCs require additional signals to CCL21/CCR7 to access the LN parenchyma: in cutaneous allergic responses, CD301b+ DCs were found to require CCR8 signaling in response to CCL8 from interfollicular CD169+SIGN-R1+ macrophages (99). Within the LN parenchyma, chemokines from a variety of cellular sources exquisitely regulate localization of specific leukocyte subsets to coordinate effective immune responses, reviewed in detail elsewhere (49, 85).

In response to infection and inflammation, LN LECs respond robustly and dynamically. Proliferation of LECs supports the expansion of LNs during immune responses and coincides with increased expression of chemokines including CXCL9, CXCL10, CCL2, CCL5, and CCL20 (31, 32, 100) (Table 2). In the latter stages of inflammatory remodeling of the LN, the cortical and medullary sinuses expand substantially (31, 103). During certain infections, such as persistent infection with the helminth *Heligmosomoides polygyrus*, lymphangiogenesis driven by VEGF-A and VEGF-C from B lymphocytes results in a sustained expansion of LN LECs (104). Such changes potentially support the egress of leukocytes from the inflamed LN and the restoration of homeostasis, however may also influence lymphoid tissue functions in response to subsequent infections.

**Table 2:** Chemokines and receptors differentially expressed in physiological and disease-associated LECs.

**Disease/tissue setting**	**Species**	**Ligands**	**Receptors**	**Atypical receptors**	**Reference**
Pre-collecting LEC (podoplanin^low^) vs. initial LEC (podoplanin^high^)	Human	↑ CCL27, CXCL12, CXCL14 ↓ CCL21	↑ ND ↓ ND	↑ ACKR1 (DARC) ↓ ND	(27)
LN subcapsular sinus vs. medullary lymphatic sinus LECs	Mouse	↑ CCL12, CXCL16, CCL25 ↓ CCL19, CCL21a, CCL21b, CXCL4 (PF4)	↑ CCR8, CXCR6 ↓ ND	↑ ACKR4 (CCRL1) ↓ ACKR1 (DARC)	(95)
Contact hypersensitivity inflamed ear skin vs. normal ear skin LECs	Mouse	↑ CXCL9, CXCL5, CXCL10, CXCL2, CCL12, CXCL14, CCL8, CCL2, CCL7, CCL9, CCL19, CXCL1, CXCL12 ↓ ND	↑ ND ↓ ND	↑ ND ↓ ND	(101)
T241/VEGF-C sarcoma vs. normal skin LECs	Mouse	↑ ND ↓ CXCL1, CXCL5	↑ ND ↓ ND	↑ ND ↓ ND	(102)
Herpes simplex virus-1 draining LN (day 6) vs. normal LN LECs	Mouse	↑ CCL21a, CCL7, CCL2, CCL5, CCL7, CCL20, CXCL9, CXCL10, CXCL13 ↓ CXCL1	↑ ND ↓ ND	↑ ACKR2 (D6, CCBP2) ↓ ND	(31)
Type 2 Diabetes vs. normal dermal LECs	Human	↑ CXCL10 ↓ CCL27, CXCL14	↑ ND ↓ ND	↑ ND ↓ ND	(28)
Afferent sentinel LN collecting lymphatic of footpad gastric tumor vs. normal	Rat	↑ CXCL14, CXCL1, CCL7 ↓ ND	↑ ND ↓ ND	↑ ND ↓ ACKR2 (D6, CCBP2)	(38)

*Results derived from microarray studies of LECs isolated from in vivo settings. ↑ relatively higher, ↓ relatively lower mRNA expression in first vs. second disease/tissue setting. ND, none detected/none described*.

Egress of leukocytes from LNs occurs predominately via the medullary lymphatic sinuses, which channel cells into efferent collecting lymphatics (49, 86). Leukocyte retention in or egress from a LN is regulated by a balance of directional signals and changes in receptor expression (49). Prolonged signaling through CCR7 in T cells or CXCR5 in B cells leads to reduced expression or responsiveness of these receptors to their ligands expressed by LN LECs or other stromal cells, and leukocytes instead upregulate S1P receptor 1 (S1PR1), the main receptor promoting leukocyte egress (105–107). Notably, LECs have been defined to be the key cellular source of S1P regulating lymphocyte egress, as determined by conditional gene deletion of the two enzymes responsible for S1P generation (Sphk1 and Sphk2) by LYVE-1-directed expression of Cre recombinase (108).

While many of the major LEC-expressed chemokines and receptors that regulate leukocyte trafficking have been defined, many more questions remain. It is evident that different pathological stimuli elicit expression of different suites of chemokines and receptors in LECs (31, 101). This indicates a role for LECs in trafficking context-specific subsets of leukocytes to LNs, as well as interacting with other cells within the tissue microenvironment, which remains to be explored.

## Wound Healing

Chemokine signaling plays an integral part in the healing process of various wounds including lacerations, surgical incisions, burns and skin grafts, as well as chronic diabetic and aging wounds. Wound healing is a dynamic and highly coordinated process with three distinct stages—inflammation, tissue formation, and tissue remodeling, with each stage having a distinct chemokine profile (109, 110). Inflammation is a crucial part of wound healing and despite the variety of tissue injuries that can occur, the subsequent events share a similar course (111). The aim of the initial inflammatory phase is to prevent further blood/fluid loss, protect against infection, and initiate the clearance of dead or dying cells and tissue debris (111). It begins immediately after tissue damage and is characterized by the formation of a platelet plug, the deposition of a fibrin matrix and the recruitment of neutrophils, which are the predominant inflammatory effector cells in the first 24–48 h (112). Monocytes enter the wound area 48–72 h after injury, differentiate into macrophages and play an important role in coordinating subsequent events of wound repair (111). The second stage, tissue formation, spans 2–10 days after injury, and aims to restore the barrier function of the epithelium (110, 111). Angiogenesis occurs from blood vessels at the wound edge and the newly formed capillaries, along with macrophages and fibroblasts, replace the fibrin matrix with granulation tissue, and allow for the proliferation and migration of keratinocytes across the wound surface (111, 113). In addition to angiogenesis, an adequate growth of lymphatic vessels in and around the wound zone is critical for the normal healing. Lymphangiogenesis follows angiogenesis via sprouting from existing lymphatic vessels at the wound edge and is primarily stimulated by VEGF-C or VEGF-D secreted by macrophages located in the microenvironment (114–116). This facilitates the drainage of tissue edema and transport of DCs from the wound zone (114, 117–119). Macrophages also stimulate some fibroblasts to differentiate into myofibroblasts and working together with fibroblasts, a predominately type III collagen extracellular matrix is deposited and the edges of the wound are brought together over time (120, 121). The final stage of tissue remodeling begins 2–3 weeks after injury and can take over a year to complete (111). It is characterized by the progressive cessation of the inflammatory response and the remodeling of the type III collagen matrix to type I collagen (122).

Although the three phases of wound healing are distinct, the inflammatory reaction continues until tissue remodeling, albeit with changing cellular mediators of inflammation (113). Leukocytes have the dual role of acting as immunological effector cells as well as modulators of inflammation. In the acute phase, the production of proteases and reactive oxygen species aids with tissue degradation, while the secretion of growth factors in the later stages promotes tissue formation (113). Chemokines are integral in activating and recruiting leukocytes to specific microanatomical sites of the wound as well as stimulating angiogenesis (63, 109). Neutrophils, the initial responders of the acute inflammatory response, are recruited by CXCL1, CXCL5, CXCL7, and CXCL8 (formerly IL-8), secreted by activated platelets, BECs, pericytes and resident monocytes within the injured tissue (123–126). Monocyte and macrophage recruitment follows closely behind and is mediated by CCL2 secretion (63). CCL2 is also chemotactic for lymphocytes but after day 4 post-injury, CXCL9, CXCL10, and CCL22 secreted by monocytes and macrophages take over (127). The role of the CXC family of chemokines in angiogenesis is well-established (63) and the concentration of CXCL1, CXCL8, and CXCL12 in the healing wound is greatest during days 1–4 post-injury and correlates with an increasing number of blood vessels within the wound (113). The high levels of CXCL1 and CXCL8 within the wound also stimulate keratinocytes via CXCR2 to increase proliferation and migration, which enhances re-epithelialization (128, 129).

While the mechanisms of wound repair (111) and the chemokines involved (109, 113) have been extensively reviewed, the effect of these chemokines on the lymphatic vasculature and the role of chemokines secreted by the lymphatic endothelium on the healing wound are not well-established. The extent to which lymphatics will respond to chemokines secreted during the various stages of wound healing will largely depend on their expression of chemokine receptors. Like BECs, LECs express receptors CXCR1 and CXCR2, both of which are upregulated during inflammation (48, 130). As such, LECs have the potential to interact with the CXCL1, CXCL5, CXCL7, and CXCL8 chemokines that are expressed in the healing wound. Of these, CXCL1 and CXCL8 have been shown to promote lymphangiogenesis via increased LEC migration and tube formation, and additionally increased LEC proliferation in the case of CXCL8 (38, 48, 131). Interestingly, CXCR2 expressed on the surface of lymphatics acts as a scavenging receptor, capable of binding various inflammatory chemokines that can shape chemokine gradients. LEC-expressed CXCR2 is thus likely to influence the inflammatory response, and has also been shown to be important in lymphatic vessel remodeling, two key components of wound healing. For example, CXCR2 ligands, CXCL1 and CXCL2, have been shown to be elevated during the inflammation stage of wound healing and in skin graft wounds (109, 132). LECs also have the ability to differentially secrete CXCL1 and CXCL8 depending on the local environment, which is thought to act in an autocrine and/or paracrine manner to increase lymphangiogenesis (133). However, in the context of a healing wound, LEC secretion of these chemokines may also have an endocrine effect in creating a chemokine gradient to recruit distant neutrophils. Another lymphangiogenic chemokine present in the healing wound is CXCL12, which binds CXCR4 expressed by LECs to induce migration and tube formation in a novel pro-lymphangiogenic pathway that is distinct from the classical VEGFR-3 pathway (46). Furthermore, increased secretion of CCL21 by LECs in the inflammatory wound environment may increase migration of DCs and other antigen-presenting cells to help activate an immune response, which may assist in healing of infected wounds (88, 101, 134).

Complications in wound healing impair the ability of lymphatic vessels to regenerate and repair, leading to impaired lymphatic drainage which results in lymphedema, with the risk for recurrent infection. Therefore, there is a clinical need to better understand the regulation of lymphatic vessel function during wound healing. Chemokines have been a focus for therapeutic approaches to promote wound healing, in particular targeting the CXCL12/CXCR4 signaling axis, a key pathway regulating the recruitment of bone-marrow derived stem cells with regenerative capacity (135). Greater understanding of the specific chemokine pathways involved in wound healing will present additional therapeutic opportunities.

## Cancer

While cancer is a genetic disease initiated through the acquisition of specific mutations in key genes, the resulting changes to the cell biology within a host drives the important clinical manifestations of the disease. The progression of cancer, from its evasion of the immune system to its ultimate spread to critical organs systems in the body, has a reliance on altered chemokine signaling resulting from the presence of mutated tumor cells. The lymphatics play a key role in both controlling access to and interaction with the immune system, and also provide an initial means of escape for primary tumor cells, while chemokines also influence immune responses and the pattern of metastatic spread through directed migration of tumor cells in a tissue-specific manner (11, 17, 34, 136).

### Leukocyte Recruitment and Egress

Multiple chemokines and receptors have been implicated in the recruitment of specific immune cell subsets to tumors and in influencing cancer immunotherapy responses (26, 137). However, the involvement of lymphatics in anti-tumor immune responses is only beginning to be understood. Powerful lymphangiogenic growth factors that drive the formation and remodeling of lymphatic vessels have been shown to upregulate chemokines in the context of cancer. VEGF-C has been shown to upregulate CCL21 expression by LECs, driving CCR7-dependent tumor chemoinvasion toward lymphatic vessels (138). CCL21 has also been shown to promote lymphoid-like stromal components and immune escape in melanoma tumors in mice, raising the concept that CCL21-secreting tumors can alter the host immune response from immunogenic to tolerogenic which then impacts on tumor progression (139). Recent extension of these observations has shown a role for VEGF-C-induced CCL21 in the tumor infiltration of naive T cells prior to immunotherapy via CCR7-dependent chemotaxis (140). The authors of this study propose that VEGF-C, through VEGFR-3 signaling, can potentiate immunotherapy by attracting abundant CCR7+ naive T cells, which are then locally activated by the immunotherapy. These studies point to a role for VEGF-C and potentially other lymphangiogenic factors as predictive biomarkers for immunotherapy, with a chemokine providing a key link in the signaling chain (140). Meanwhile, other studies are beginning to unravel the complex mechanisms by which lymphatics influence the tumor immune microenvironment (141, 142). Other groups have speculated that “key driver chemokines”—for example CXCL10, which is expressed by LECs in several pathological contexts and implicated in metastasis to LNs (28–30, 143)—may be valid targets in diseases including cancer because of their ability to enhance T-cell-dependent anti-cancer immunity (144).

### Tissue-Specific Patterns of Metastasis

A common manifestation of chemokine involvement in directing patterns of metastasis is that tumor cells express chemokine receptors that respond to chemokines secreted by cells of a given tissue or organ, often co-opting the chemokine signaling used for tissue-specific homing of leukocytes (11, 26, 34, 35). This is true for lymphatic vessels and LECs present in the primary tumor, regional LNs or distant organs that are targets of metastatic spread. Studies in a variety of tumor types have shown that CCR7 present on the tumor cells mediates their migration toward CCL21-expressing initial lymphatics (in preference to blood vessels) and/or LNs, thereby promoting spread via the lymphogenous route (145) [recently reviewed in (11, 26)]. A similar mechanism is mediated by tumor-expressed CXCR4 and CXCL12 expressed in LECs and LNs (11, 26). In particular, CXCL12 secreted by LECs in the LN SCS contributes to an attractive and supportive metastatic niche for CXCR4+ tumor cells (36, 37).

The list of chemokines that similarly promote LN metastasis is expanding. Expression of CXCR3 in colorectal cancer was linked to increased metastasis to LNs, likely in response to expression of ligands CXCL9, CXCL10, and CCL21 in the lymphatic sinuses and paracortex of LNs (29). As the rate of spread of CXCR3 -expressing or -deficient cell lines was similar, potential growth effects were also considered. A similar effect was also seen in melanoma (30). In gastric cancer, LECs upregulated expression of CXCL1, which in turn increased tumor cell invasiveness via CXCR2 and stimulated lymphangiogenesis. Expression of both ligand and receptor in patient gastric cancer specimens was associated with LN metastasis and poor survival (38, 39). Soler-Cardona et al. characterized a mechanism in melanoma where neutrophils recruited to melanomas by CXCL5 appeared to facilitate transmigration of tumor cells through lymphatic endothelium (47). Expression of CCL1 in the LN SCS was also shown to regulate entry of CCR8+ melanoma cells into the LN (43). It is noteworthy that recent studies in mouse models have shown that initial lymphogenous spread can transfer to the blood vascular system via high endothelial venules within regional LNs (146–148).

With the exception of their arrival to LNs via afferent lymphatics, tumor cells are presumed to home to specific organs via the blood vessels, guided by chemokines produced by BECs or transcytosed to the vessel lumen (11, 34). Nonetheless, LECs in distant organs can also participate in distant organ metastasis and metastatic niche formation. Lee et al. showed in mouse models that circulating IL-6 secreted by orthotopic breast cancer cells could influence LECs in LNs and lung. These distant LECs were induced to express CCL5, which was chemotactic for the tumor cells, and VEGF-A, which increased angiogenesis and vascular permeability at metastatic sites (42). Another study used a *Vegfr3*-luciferase reporter mouse and melanoma models to demonstrate pre-metastatic lymphangiogenesis in distant organs, and to identify midkine as a regulator of metastatic niche formation with prognostic significance (149). These studies open up new avenues of investigation into how lymphatics at distant metastatic sites can also influence metastasis.

### Atypical Chemokine Receptors in Cancer

Atypical or decoy chemokine receptors have in a number of contexts been involved in modulating cancer progression through shaping the inflammatory response (7, 150). The atypical chemokine receptor ACKR2 has been shown to internalize and sequester an array of pro-inflammatory chemokines of the CC family (5, 49, 50). Mice deficient in ACKR2 had an increased susceptibility to the development of cutaneous tumors that was linked to the recruitment of immune cells (e.g., T cells and mast cells) to support their development (54). In this study ACKR2 was predominately expressed in LECs of human oral squamous cell carcinomas (OSCC) and not tumor cells or epithelial cells, and the levels in tumor LECs were upregulated compared to normal LECs (54). This same group had previously shown that ACKR2 is expressed by lymphatic endothelium and may influence the recirculation of leukocytes via a chemokine driven mechanism, as ACKR2 was expressed on the afferent lymphatics (51). Antigen-experienced T cell subsets express multiple CCR receptors, with CCR4 specifically implicated in cutaneous T cell homing (151). Mast cells also express CCL3 receptors, and these cells generally play a role in promoting tumor angiogenesis and recruiting other pro-tumorigenic leukocyte subsets. CCL3 further directly contributes to mast cell degranulation via CCR1 (152). Expression of ACKR2 therefore limited inflammation by restricting availability of chemokines that attracted these pro-inflammatory leukocytes (54). Other studies of ACKR2 have confirmed its role in the lymphatic system in other organs. ACKR2-deficient mice are more susceptible to inflammation-induced colon carcinogenesis, an effect that was attributed to lymphatic expression of ACKR2 using bone marrow transplantation experiments (55). ACKR2 was also found to be upregulated on lymphatics of inflamed and cancerous colon specimens (55). However, other groups have reported contrasting results (56, 57), potentially suggesting dynamic and context-specific roles for ACKR2 during inflammatory colon carcinogenesis. ACKR2 is also highly expressed in vascular tumors of lymphatic origin (51) and the spindle cells of Kaposi's sarcoma (58). In more aggressive tumors this receptor is down-regulated through the KRAS/BRAF/ERK pathway, leading to chemokine-mediated macrophage recruitment and increased angiogenesis and tumor growth (58).

In breast cancer the absence of a number of members of the atypical chemokine receptor subset predict involvement of axillary LN metastasis, a key clinicopathological indictor of disease progression (153). These observations were further validated by the characterization of genetic variants of two chemokine decoy receptors, ACKR1 and ACKR2, that associated with the metastatic potential of breast cancer (154, 155). Yu et al. found that the expression of the atypical chemokine receptors also predicted relapse-free survival in breast cancer where co-expression and co-genotype (two major alleles of DARC-rs12075 and D6-rs2228468) of the chemokine decoy receptors ACKR1 (DARC) and ACKR2 (D6) had significant associations. This data shows that host factors such as polymorphisms of major chemokine receptor genes and the expression of the protein receptors in cancers, including in lymphatic or blood vessels, could help predict prognosis (155).

### Tumor Lymphangiogenesis

Lymphangiogenesis and lymphatic remodeling in tumors, commonly driven by VEGF-C and VEGF-D, are strongly associated with metastasis to LNs and distant organs (17). We recently identified a cooperative role for CCL27, CCL28, and their receptor CCR10 in VEGF-D driven tumor lymphangiogenesis (44). Here, CCR10 was expressed by LECs and upregulated by VEGF-D and the pro-inflammatory cytokine TNF-α. LECs were attracted to both CCL27 and CCL28 in a CCR10-dependent fashion. Further examination of CCR10-deficient mice confirmed a role for this receptor in lymphatic patterning. While CCL27 alone was not sufficient to drive metastasis, both chemokines enhanced LEC migration and worked in combination with VEGF-D to recruit LECs and form coherent vessels (44). The study suggests a cooperative action of chemokines, inflammatory mediators, and lymphangiogenic growth factors during cancer progression. Interestingly, VEGF-D was also shown to upregulate expression of ACKR2 in LECs *in vitro* (53). Other studies have shown a link between chemokine signaling and VEGFR-3-driven lymphangiogenesis in cancer where VEGF-C can upregulate CXCR4 and thereby cooperate with CXCL12 in driving lymphangiogenesis and metastasis (46). Notably CXCR4 has a well-established role in tumor angiogenesis as well, and is being actively pursued as a therapeutic target (64, 156). Tumor-expressed CXCL5 in melanoma has also been found to drive tumor lymphangiogenesis and lymphogenous metastasis through CXCR2 expressed on LECs (47). Gastric cancer cells induce expression of CXCL1 in LECs, which subseqently drives tumor lymphangiogenesis and lymphogenous metastasis (38). These studies illustrate that as well as being a source of chemokine ligands in cancer, lymphatic vessels can also be guided by chemokine receptor signaling.

Lymphangiogenesis is also coupled with chemokine signaling by fluid mechanics (157). Lymphatic flow is important in stimulating chemokine secretion by LECs and other cells, as well as for generating gradients of chemokines that can be followed by migrating tumor cells (98, 157, 158). Under conditions of interstitial flow, tumor cells co-expressing a chemokine and its receptor can thereby exhibit “autologous chemotaxis,” following a self-generated chemokine gradient toward lymphatics (159).

## Other Pathologies

Lymphatic vessels have been observed to intersect with chemokine-mediated movement of important effector cells in a variety of diverse human pathologies. In type 2 diabetes patients a range of chemokines and related genes were differentially expressed in dermal LECs compared to non-diabetic patient LECs (28) (Table 2). Enhanced lymphatic density was observed in skin, along with upregulation of CXCL10 and downregulation of CCL27 and CXCL14 in response to pro-inflammatory conditions. TNF-α upregulated CXCL10 in LECs, and LEC-derived CXCL10 was able to mediate macrophage adhesion to LEC monolayers and invasion into agarose plugs (28). The study identified paracrine cross-talk allowing macrophage recruitment toward LECs via a chemokine-mediated mechanism.

Studies of the mechanisms of human kidney transplant rejection show that inflammatory infiltrates rich in lymphocytes attack both cortical tubules and endothelial cells. This is accompanied by significant increases in local lymphatic vessel density due to “lymphatic neoangiogenesis” (94). LECs from these vessels express and secrete CCL21 which attracts CCR7+ cells (94). A later study showed that ACKR3 (CXCR7) was also expressed by LECs during kidney rejection with nearly 1/3 of adult dermal lymphatics expressing ACKR3, and both ACKR3+ blood and lymphatic vessels increasing in number during allograft rejection (59).

A role for ACKR2 on lymphatic endothelium in autoimmune disease is implied from studies of the ACKR2-deficient mice during experimental autoimmune encephalomyelitis (EAE) (160) where encephalitogenic responses, including DC migration and T cell priming, were impaired (160). Interestingly other studies have shown that ACKR2-deficient mice develop enhanced symptoms of EAE (as well as collagen-induced arthritis) due to enhanced Th17 responses (161). These differences could be due to control of IL-17 production by ACKR2 (50).

## Future Directions and Conclusions

Lymphatic vessels, like blood vessels, are a highly interactive surface for cells of the immune system, and through the use of chemokines and their receptors can coordinate key interactions. These pathways can control the entry and function of particular immune subsets in a number of pathological conditions. Nonetheless LECs have distinct patterns of chemokine secretion and expression of chemokine receptors that distinguish them from the blood vessel system and mediate distinct roles and responses. The abundance and diversity of the chemokine family point to the likelihood that a plethora of novel chemokine functions and interactions remain to be discovered. Of note, several recent studies have undertaken differential expression profiling of LECs by microarray in a range of different pathologies, revealing multiple chemokines with as-yet undefined roles in disease (Table 2). These studies are complemented by *in vitro* analyses examining chemokines and receptors upregulated in LECs by specific stimuli (46, 60, 88). The emerging data suggests that chemokines and their receptors play a complex role in helping coordinate the movement of LECs and interactive circulatory cells in both normal development and a range of pathological conditions.

## Author Contributions

RF and SS conceived the review. RF, TK, SiM, ScM, and SS wrote and edited the manuscript.

### Conflict of Interest Statement

SS has ownership interest in Opthea Ltd. that develops therapeutics in vascular biology. The remaining authors declare that the research was conducted in the absence of any commercial or financial relationships that could be construed as a potential conflict of interest.

## Abbreviations

BEC: Blood vascular endothelial cell
DC: Dendritic cell
LEC: Lymphatic endothelial cell
LN: Lymph node
LTi: Lymphoid tissue initiator
LTo: Lymphoid tissue organizer
SCS: Subcapsular sinus (of lymph node).
